# Adherence to *a priori* and a posteriori dietary patterns and risk of Parkinson’s disease: a systematic review and meta-analysis of observational studies

**DOI:** 10.3389/fnut.2025.1600955

**Published:** 2025-05-12

**Authors:** Rong Zhang, Long Shu, Qin Zhu, Nan Li

**Affiliations:** ^1^Department of Digestion, Zhejiang Hospital, Hangzhou, Zhejiang, China; ^2^Department of Clinical Nutrition, Zhejiang Hospital, Hangzhou, Zhejiang, China

**Keywords:** Parkinson’s disease, dietary patterns, systematic review, meta-analysis, observational studies

## Abstract

**Background:**

Although studies have reported the associations between certain dietary patterns and the risk of Parkinson’s disease, these findings are limited and inconclusive. Herein, we carried out a systematic review and meta-analysis of observational studies to search for the associations between *a priori* and a posteriori dietary patterns and the risk of developing Parkinson’s disease.

**Methods:**

We systematically searched PubMed, Web of Science, Scopus, and China National Knowledge Infrastructure from database inception to January 2025 to clarify eligible observational studies investigating the links between whole dietary patterns and risk of Parkinson’s disease. Combined relative risks (RRs) and 95% confidence intervals (CIs) were calculated for the highest versus lowest categories of dietary patterns in relation to Parkinson’s disease risk. The Cochran’s *Q* test and *I*-squared (*I^2^*) statistic were used to assess statistical heterogeneity among the included studies.

**Results:**

In total, 11 studies (five cohort, three case–control, and 3 cross-sectional studies) with 326,751 participants and 2,524 cases were included in this meta-analysis. The pooled analyses showed that adherence to the Mediterranean diet, healthy dietary index, and healthy dietary pattern were associated with a decreased risk of Parkinson’s disease (RR = 0.87; 95%CI: 0.78–0.97, *p* = 0.017; RR = 0.76; 95%CI: 0.65–0.91, *p* = 0.002; RR = 0.76; 95%CI: 0.62–0.93; *p* = 0.007, respectively). Additionally, the results showed that high adherence to the Western dietary pattern was associated with an increased risk of Parkinson’s disease (RR = 1.54; 95%CI: 1.10–2.15; *p =* 0.011).

**Conclusion:**

Overall, our results demonstrate that adherence to the Mediterranean diet, a healthy dietary index, and a healthy dietary pattern were associated with a reduced risk of Parkinson’s disease, while the Western dietary pattern was linked to an increased risk of Parkinson’s disease. Further well-designed prospective studies and randomized controlled trials are required to confirm these findings.

## Introduction

Parkinson’s disease is a common and progressive neurodegenerative disease, affecting approximately 6.1 million people worldwide (1). In the United Kingdom, the prevalence of Parkinson’s disease was estimated at 286.5 per 100,000 person-years in 2020 (2). Notably, studies have reported that the incidence of Parkinson’s disease increases with age, and is generally higher among White person/persons/people than among Black person/persons/people or Asians (3). In the United States, the economic burden of Parkinson’s disease is expected to increase from $52 billion in 2017 to $79 billion in 2037 (4). Currently, there is no curative therapy for Parkinson’s disease, so effective strategies for the prevention or delay of disease occurrence are needed (5). As far as we know, the exact cause of Parkinson’s disease remains uncertain, and it may involve the combined effects of various factors, including genetic predisposition, ageing and environmental exposures (6). Among environmental factors, dietary factors have been considered as the important modifiable factors for the prevention of Parkinson’s disease (7).

In past several decades, previous epidemiological studies have explored the potential correlations between intakes of individual foods, nutrients, or food groups and the risk of developing Parkinson’s disease, with inconsistent results (8–10). However, due to the complexity of the diet and potential interactions between food components (11), these studies showed a limited effect of diet on Parkinson’s disease. In reality, people usually eat foods that contain multiple combinations of foods and nutrients (12). As a result, dietary pattern analysis has emerged as a valuable approach and been widely used in nutritional epidemiology, taking into account the combined effects of foods and nutrients (13).

Currently, little is known about the role of whole dietary patterns in Parkinson’s disease risk. To date, limited observational studies have reported the associations between *a priori* and a posteriori dietary patterns and Parkinson’s disease risk (14–24). However, results from these previous studies have been inconsistent. Whilst some observational studies have shown the potential neuroprotective effect of adherence to healthy dietary patterns (e.g., Mediterranean diet and prudent pattern) on Parkinson’s disease (14–16, 18, 22), other studies did not find such association (17, 21, 23). For example, a case–control study reported an inverse association between higher Mediterranean-type diet adherence and Parkinson’s disease risk (OR = 0.86; 95%CI: 0.77–0.97) (15). By contrast, in a prospective population-based cohort Study, Strikwerda and colleagues found no significant association between adherence to the Mediterranean diet and Parkinson’s disease risk (HR = 0.80; 95%CI: 0.50–1.29) (20). Notably, a recent systematic review and meta-analysis of 12 observational studies found that high adherence to the Mediterranean diet was associated with a lower risk of Parkinson’s disease (25). But, in the aforementioned meta-analysis, the outcome of interests included Parkinson’s disease, prodromal Parkinson’s disease and two of included studies reported the impact of Mediterranean diet on prodromal Parkinson’s disease features. Additionally, Zhao et al.’s meta-analysis included a case–control study of healthy pattern (identified by factor analysis) and Parkinson’s disease (16) and thus had methodological limitations. Furthermore, to our knowledge, there are no systematic review and meta-analyses that have comprehensively assessed the associations between a posteriori and *a priori* dietary patterns and risk of Parkinson’s disease. Therefore, to determine the potential correlations between whole dietary patterns and risk of Parkinson’s disease, we conducted a systematic review and meta-analysis to summarize the available evidence from observational studies published up to January 2025.

## Methods

### Search strategy and selection criteria

A comprehensive literature search in four online electronic databases (PubMed, Web of Science, Scopus, and China National Knowledge Infrastructure) was performed for pertinent articles published up to January 2025, with the predefined search terms and keywords: (“dietary pattern” OR “eating pattern” OR “food pattern” OR “diet indices” OR “dietary score” OR “dietary quality” OR “dietary index” OR “diet”) AND (“Parkinson’s disease” OR “Parkinson disease” OR “Parkinsonism”). No publication date or language restrictions were applied during the search process. In addition, the reference lists of all eligible articles or reviews were manually searched to identify any additional relevant citations to ensure a comprehensive search. This meta-analysis was performed in accordance with the Preferred Reporting Items for Systematic Reviews and Meta-Analysis (PRISMA) statement (26). The protocol for this systematic review and meta-analysis was developed, but not registered online in advance with the International Prospective Register of Systematic reviews (PROSPERO) database. The literature search was independently performed by two of the authors (R.Z. and L.S.). Any discrepancies in searching articles were resolved by consensus or by consulting a third author (N.L.). Details of search strategy have been shown in the Supplementary Table 1.

To be included in this review, the eligible articles met the following criteria: (1) observational research (e.g., cohort, case–control, cross-sectional study) performed in humans of any age; (2) studies exploring the correlations between whole dietary patterns and risk of Parkinson’s disease; (3) dietary patterns were identified using a posteriori methods (e.g., factor analysis or cluster analysis) or *a priori* methods; (4) provided risk estimates of ORs, RRs, HRs along with their corresponding 95%CIs; (5) Parkinson’s disease diagnosis was confirmed by medical records review or a neurologist; (6) if retrieved article lacked sufficient Parkinson’s disease-relevant data, the corresponding author of original study will be contacted for additional information by email.

Studies were excluded based on the following criteria: (1) unrelated articles, e.g., retrieved articles did not report the association between diet and risk of Parkinson’s disease; (2) non-observational studies, e.g., intervention studies, reviews, editorials, case reports and conference letters; (3) grey literature, which is generally not included in large electronic databases, such as PubMed, Web of Science, Scopus and China National Knowledge Infrastructure; (4) Parkinson’s syndrome instead of Parkinson’s disease; (5) studies not reporting HRs, RRs or ORs with 95% CIs. Two authors (R.Z. and L.S.) independently examined all the titles and abstracts, and obtained full texts of potentially relevant articles. Any disagreements about including eligible studies were addressed through discussion or, if need, in consultation with the third author (N.L.). Our selection criteria was based on the PECOS (e.g., participant, exposure, comparison, outcome, and study design) framework, which is shown in Table 1.

**Table 1:** PECOS criteria for inclusion and exclusion of studies.

Parameter	Criteria
Population	Adults (≥18 years)
Exposure	Dietary patterns (index-based or data-driven)
Comparator	Highest vs. lowest categories of exposure
Outcomes	Parkinson’s disease
Study design	Observational studies (prospective cohort, case–control or cross-sectional)

PECOS, population, exposure, comparator, outcome and study design.

To minimize error, we ensured that dietary patterns chosen were similar in terms of the factor loads of foods consumed in those dietary patterns. For example, Mediterranean diet is characterized by high consumption of fruits, vegetables, nuts, legumes, whole grains and extra-virgin olive oil; moderate consumption of poultry, fish and alcohol; and low consumption of red and processed meats (15). Healthy dietary pattern is characterized by high consumption of fruit, vegetables, legumes, whole grains, poultry, and fish (14). Western dietary pattern is characterized by high consumption of red and/or processed meat, refined grains, French fries, sweets, desserts and high-fat dairy products, and low consumption of fruits and vegetables (14).

### Data extraction

For each eligible article, two independent authors (R.Z. and L.S.) extracted the following data using a standardized form: first author’s name, publication year, study design, country where the study was performed, number of participants, number of Parkinson’s disease cases, mean age or age range (year), dietary assessment method, reported risk estimates (HRs/ORs/RRs) and the corresponding 95%CIs and confounding factors that were adjusted for in the multivariate analyses. Any disagreements about data extraction were resolved through consensus or discussion with a third author (N.L).

### Quality assessment of included studies

For non-randomized studies, the quality of eligible study was evaluated using the Newcastle-Ottawa Scale (NOS) (27). This scale assigns 0 ~ 9 “stars” to each study based on three aspects: selection of participants (maximum of 4 stars), comparability of study groups (maximum of 2 stars), and ascertainment of outcomes of interests (maximum of 3 stars). Studies with NOS scores ≥ 7 points were deemed to be of high quality (28).

### Data synthesis and statistical analysis

In this study, the reported ORs from case–control and cross-sectional studies were converted to RRs using the following formula: RR = OR/[(1-P_0_) + (P_0_*OR)], where P_0_ represents the incidence of the outcome of interest in the non-exposed group (29). In addition, HRs were considered as approximations of RRs. Statistical heterogeneity among the studies was evaluated using the Cochran’s *Q* test and *I*-squared (*I^2^*) statistics. If *p* values of Cochran’s *Q*-test ≤0.10 or *I*^2^ ≥ 50% indicated substantial heterogeneity among studies, and a random-effects model (DerSimonnian and Laird method) was used to pool the RRs and 95%CIs of the highest versus the lowest categories of priori and posteriori dietary patterns in relation to Parkinson’s disease. Otherwise, a fixed-effect model is adopted (30). To explore the possible sources of observed heterogeneity across studies, we performed subgroup analyses by study design (cohort or case–control/cross-sectional studies), study area (Western countries or other countries), mean age (≥50y or <50y), sample size (≥5,000 or <5,000), study quality (≥7 or <7) and dietary assessment method (FFQ or other/24 h dietary recall). A sensitivity analysis was also performed by removing one study at a time, and to test if the observed associations were robust or sensitive to the influence of each individual study. Publication bias was assessed through the visual inspection of the funnel plots and quantified by Begg’s and Egger’s tests (31). If the results showed evidence of publication bias, we used the trim and fill methods to adjust the asymmetry of the funnel plot by inferring the potentially missing studies (32). All statistical analyses were performed using STATA, version 12.0 (StataCorp, College Station, TX, USA). A 2-tailed *p-value* less than 0.05 was considered statistically significant.

## Results

### Search results

**Figure 1:**
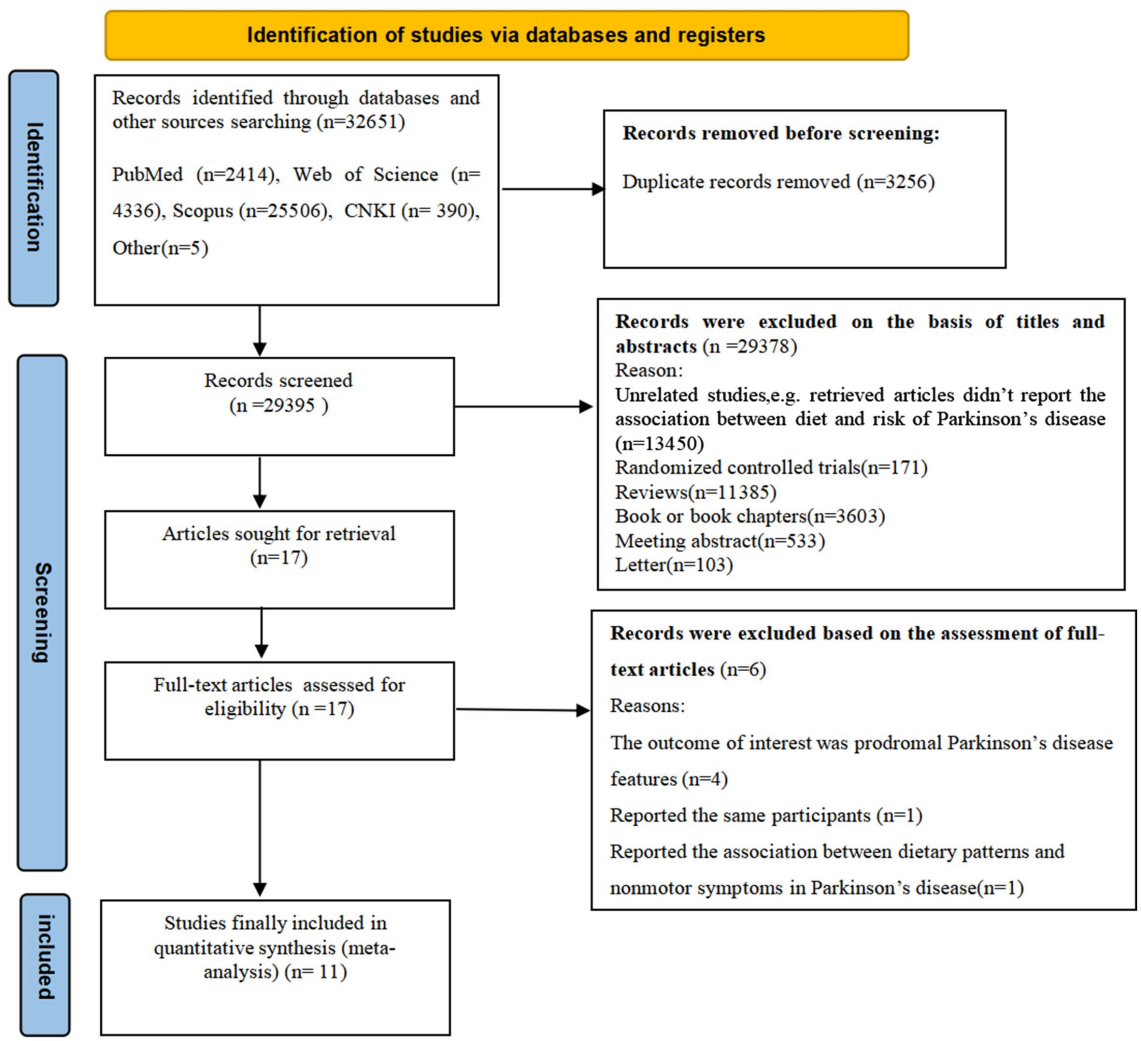
The PRISMA flow chart of literature search process.

Figure 1 illustrates the PRISMA flow chart of literature search process. Our searches initially generated 32,651 articles from four databases and other sources. After removing 3,256 duplicates, 29,395 records remained. Subsequently, 29,378 articles were removed based on the review of titles and abstracts of retrieved articles and irrelevant articles. The remaining 17 full-text articles were reviewed in details, and 6 articles were excluded for the following reasons: the outcome of interest was prodromal Parkinson’s disease features (*n* = 4); reported the same participants (*n* = 1); reported the association between dietary patterns and nonmotor symptoms (*n* = 1). Accordingly, 11 studies met the eligibility criteria and were finally included in this meta-analysis.

### Study characteristics

The characteristics of included studies were detailed in Table 2. Eleven studies, including 5 prospective cohort (14, 17, 19, 20, 24), 3 case–control (15, 16, 22) and 3 cross-sectional studies (18, 21, 23), with a total of 326,751 participants and 2,524 cases were included in this systematic review and meta-analysis. These included studies were published between 2007 and 2024. Sample size varied from 170 to 131,368. The age of participants ranged from ages 18 to above. Among these published studies, four studies were carried out in the United States (14, 15, 18, 23), two in Iran (21, 22), one in Japan (16), one in Sweden (17), one in Netherlands (20), one in Finland (24), and one study in United Kingdom (19). The majority of the included studies used FFQ to measure dietary intake (14, 15, 17, 20–23), two studies used dietary questionnaire (16, 19), one used dietary history interview (24), and one used 24-h dietary recalls (18). Finally, according to NOS tool, eight studies were considered to be of high quality (14–20, 22, 24), and the remaining two articles were identified as medium-quality (21, 23).

**Table 2:** Characteristics of included studies on the associations between a priori and a posteriori dietary patterns and Parkinson’s disease risk.

Author Publication Year	Country	Study design	Total number of participants	Mean age/age range	Dietary assessment method	Adjustment or matched for in analyses	Effect sizes OR/RR(95%CI)
Gao et al. (2007) (14)	United States	Cohort	131,368(508cases)	40–75y	FFQ	Age, smoking status, BMI, use of nonsteroidal antiinflammatory drugs, and intakes of total energy (kcal/d), caffeine (quintiles), and alcohol.	Prudent pattern: RR: 0.78 (0.56–1.07); Western pattern: RR 1.29 (0.71–2.0.34); Alternate Mediterranean Diet: RR: 0.75 (0.57–1.00); Alternate Health Eating Index: RR: 0.70 (0.51–0.94)
Alcalay et al. (2012)(15)	United States	Case–control	455 (257 cases)	45–75y	FFQ	Age, education and race.	Mediterranean diet OR: 0.48 (0.28–0.82)
Okubo et al. (2012) (16)	Japan	Case–control	617 (249 cases)	≥55y	Dietary history questionnaire	Non-dietary factors, including gender, age, region, pack-years of smoking, education, and body mass index.	Healthy pattern OR:0.54 (0.32–0.92);Western pattern: OR:01.49 (0.92–2.40).
Yin et al. (2021) (17)	Sweden	Cohort	47,128 (101 cases)	29–49y	FFQ	Year of birth, body mass index, smoking, physical activity, total energy intake, education, diabetes, and hypertension.	Mediterranean dietary pattern: HR:0.54 (0.30–0.98)
Xu et al. (2023) (18)	United States	Cross-sectional	5,824 (91 cases)	≥50y	24-h dietary recalls	Age, gender; race, energy intake, total carbohydrate, total sugars, and total vitamin B6.	Mediterranean diet OR: 0.78 (0.65–0.93); Prudent pattern OR: 1.35 (0.72–2.51); Western pattern OR 2.19 (1.16–4.14).
Tresserra-Rimbau et al. (2023) (19)	United Kingdom	Cohort	126,283 (577 cases)	40-69y	Dietary questionnaire	Sex, stratified by age and region, education, BMI, smoking, alcohol, energy intake, diet variation, multimorbidity and polypharmacy index.	Healthful plant-based diet index: HR:0.78 (0.61–0.99)
Strikwerda et al. (2021) (20)	Netherlands	Cohort	9,414 (129 cases)	≥55y	FFQ	Sex, age at baseline, Rotterdam Study cohort, body mass index, education, smoking behavior and energy intake	Mediterranean diet HR: 0.80 (0.50-−1.29);Prudent pattern HR: 0.89 (0.52–1.50); Unhealthy pattern HR: 1.08 (0.67–1.74).
Keramati et al. (2023) (21)	Iran	Cross-sectional	170 (120 cases)	40–80y	FFQ	Age, sex, BMI, caloric intake, smoking, diabetes, hypertension, thyroid disorder, cardiovascular diseases, medications, and physical activity	Mediterranean Diet: OR: 1.392 (0.392–4.947);
Shokri-Mashhadi et al. (2024) (22)	Iran	Case–control	320 (105 cases)	54–81y	FFQ	Age, sex, BMI, smoking, physical activity level, and energy intake	Healthy pattern OR:0.328 (0.153–0.704);Western pattern: OR:5.396 (2.254–12.915).
Agarwal et al. (2018) (23)	United States	Cross-sectional	706 (302 cases)	59–97y	FFQ	Age, sex, smoking, total energy intake, BMI, depressive symptoms.	Mediterranean diet HR:0.98 (0.96–1.01).
Sääksjärvi et al. (2013) (24)	Finland	Cohort	4,466 (85 cases)	40–79y	Dietary history interview	Age, sex, marital status, community density, geographical area, smoking, BMI, leisure-time physical activity, energy, hypertension, serum total cholesterol, diabetes and in addition, in women, parity.	Modified alternate healthy eating index: Men HR:1.83 (0.65–5.18); Women HR:0.97 (0.38–2.48).

BMI: Body mass index; FFQ: Food frequency questionnaire; HR: Hazard ratios; OR: Odd ratios; RR: Relative ratios.

### Priori dietary patterns and Parkinson’s disease

#### Mediterranean diet and Parkinson’s disease

**Figure 2:**
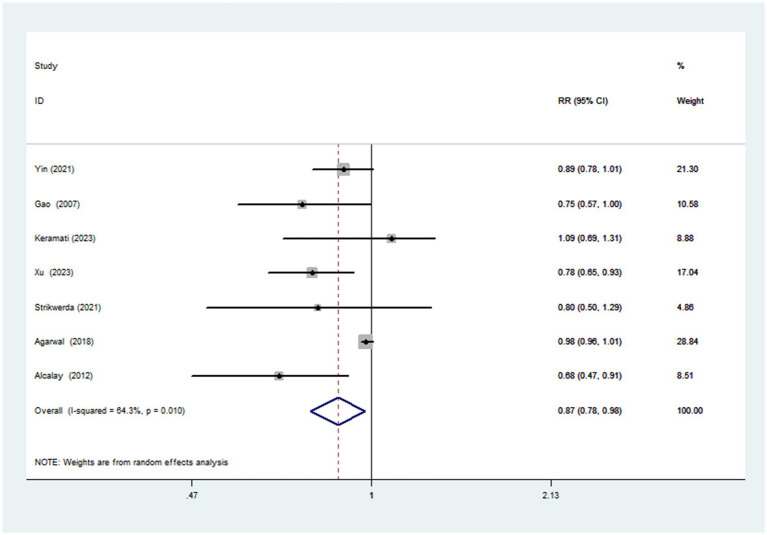
Forest plot of the association between adherence to the Mediterranean diet and risk of Parkinson’s disease.

Seven articles with a total of 195,065 participants and 1,508 cases evaluated the association between adherence to the Mediterranean diet and risk of Parkinson’s disease. Figure 2 showed the evidence of a reduced risk of Parkinson’s diseases in the highest compared with lowest categories of Mediterranean diet (RR = 0.87; 95%CI: 0.78–0.97, *p* = 0.017). Substantial heterogeneity was observed in the included studies (*I*^2^ = 64.3%, *p* = 0.010), and we used a random-effects model to calculate the pooled RRs. To explore the reasons for substantial heterogeneity across studies, we carried out subgroup analyses according to study design, study area, mean age, sample size, study quality and dietary assessment method. The results showed that study design, sample size and study quality might contribute to significant heterogeneity (Table 3).

**Table 3:** Subgroup analyses for the association between adherence to the Mediterranean diet and Parkinson’s disease risk.

Study characteristic	No. of studies	RR (95%CI)	Heterogeneity	
			*I*^2^ (%)	*P*
All	7	0.87 (0.78–0.97)	64.3	0.010
Study design				
Case–control/cross-sectional	4	0.88 (0.74–1.05)	73.1	0.011
Cohort	3	0.86 (0.77–0.96)	0.0	0.530
Study area				
Western countries	6	0.85 (0.75–0.96)	69.3	0.006
Other countries	1	1.09 (0.79–1.50)	–	–
Mean age				
≥50	6	0.85 (0.73–1.00)	66.9	0.010
<50	1	0.89 (0.78–1.01)	–	–
Sample size				
≥5,000	4	0.84 (0.76–0.92)	0.0	0.555
<5,000	3	0.92 (0.75–1.14)	60.9	0.078
Study quality				
≥7	5	0.82 (0.75–0.90)	0.0	0.482
<7	2	0.98 (0.96–1.01)	0.0	0.517
Dietary assessment method				
FFQ	6	0.90 (0.80–1.00)	54.9	0.050
24-h dietary recall	1	0.78 (0.65–0.93)	–	–

FFQ: Food frequency questionnaire; RR: relative risk; CI: confidence interval.

#### Healthy dietary index and Parkinson’s disease

**Figure 3:**
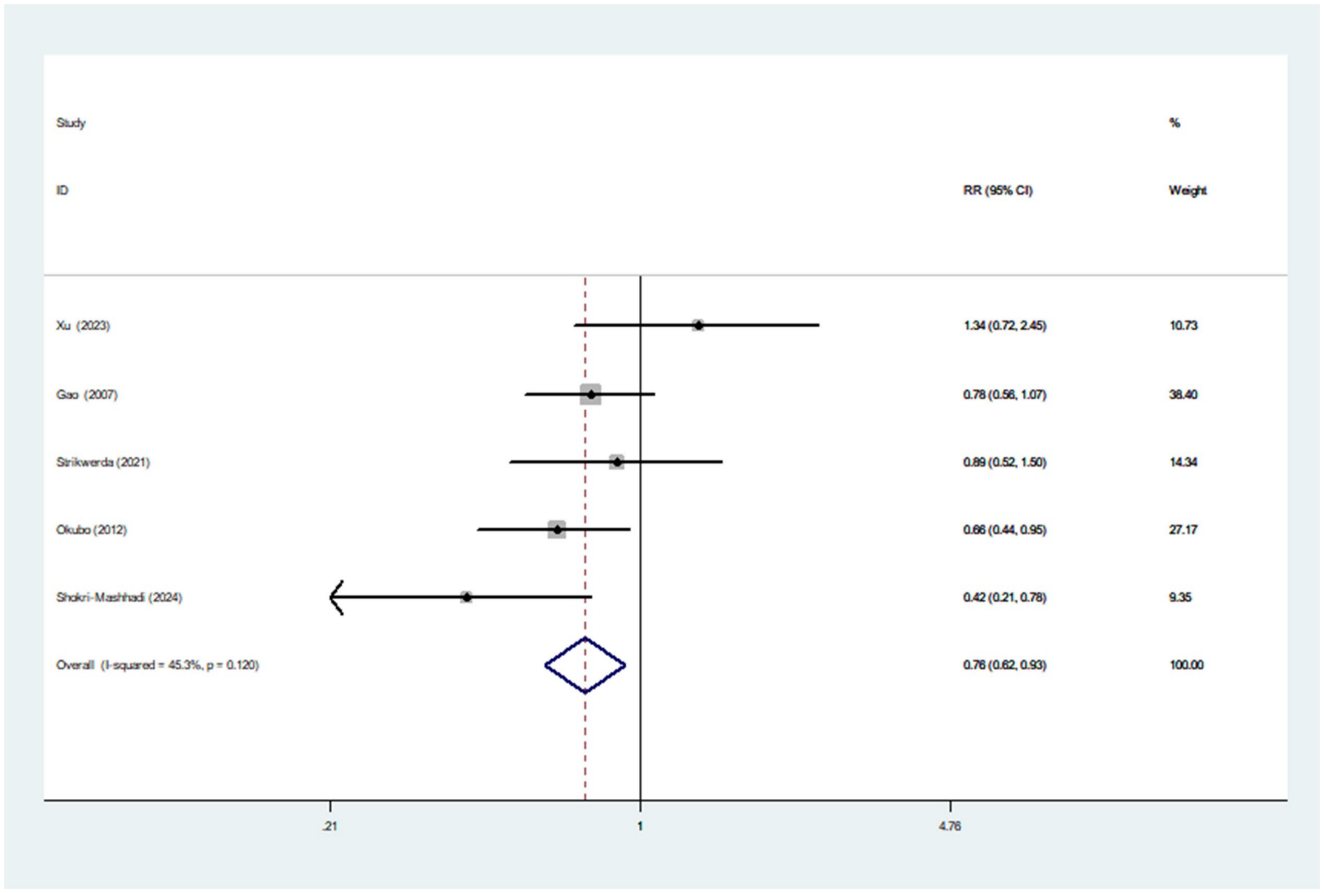
Forest plot of the association between healthy dietary index and risk of Parkinson’s disease.

Three articles incorporating four studies, were included in the analysis of healthy dietary index and the risk of Parkinson’s disease. Figure 3 showed that the highest category of healthy dietary index had a reduced risk of Parkinson’s disease (RR = 0.76; 95%CI: 0.65–0.91, *p* = 0.002) than the lowest category. Low heterogeneity between studies was found (*I*^2^ = 13.5%, *p* = 0.325). Therefore, the effect size was assessed using a fixed-effects model.

### Posteriori dietary patterns and Parkinson’s disease

#### Healthy dietary pattern and Parkinson’s disease

**Figure 4:**
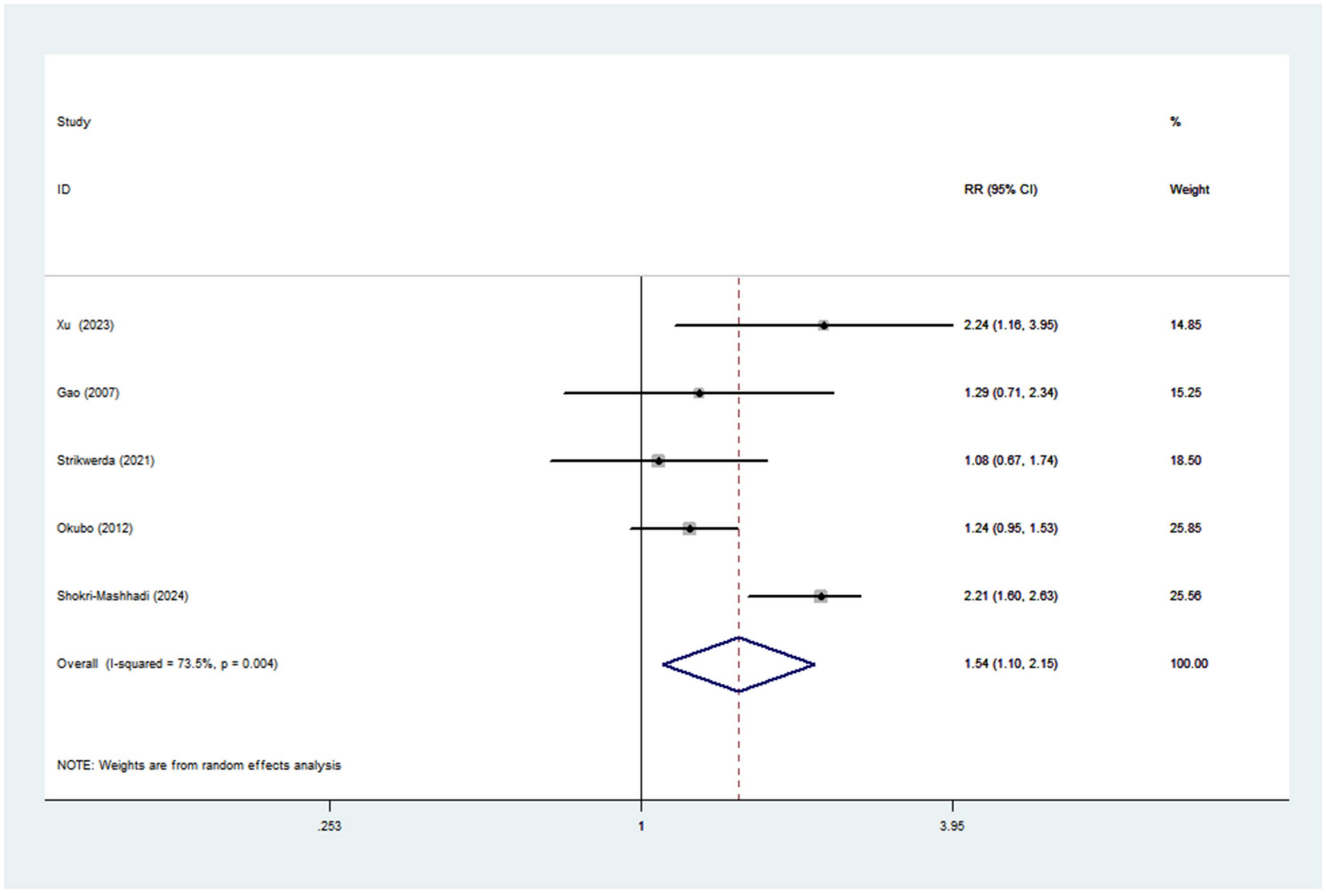
Forest plot of the association between healthy dietary pattern and risk of Parkinson’s disease.

Five articles involving 1,082 cases and 147,543 participants, were included in this meta-analysis. Figure 4 showed that the highest category of healthy dietary pattern had a 24% reduced risk of Parkinson’s disease than the lowest category (RR = 0.76; 95%CI: 0.62–0.93; *p* = 0.007). The moderate heterogeneity across studies was found (*I*^2^ = 45.3%, *p* = 0.120), and a fixed-effects model was used for data analysis. To further detect the probable sources of moderate heterogeneity, we carried out subgroup analyses depending on study design, study area, sample size and dietary assessment method. The results of subgroup analyses showed that study area and sample size might be potential source of moderate heterogeneity in included studies (Table 4).

**Table 4:** Subgroup analyses for the association between posteriori dietary patterns and Parkinson’s disease risk.

	Healthy dietary pattern					Western dietary pattern			
Study characteristic	No. of studies	RR (95%CI)	Heterogeneity		Study characteristic	No. of studies	RR (95%CI)	Heterogeneity	
			*I*^2^ (%)	*P*				*I*^2^ (%)	*P*
All	5	0.76 (0.62–0.93)	45.3	0.120	All	5	1.54 (1.10–2.15)	73.5	0.004
Study design					Study design				
Case–control/cross-sectional	3	0.71 (0.53–0.95)	70.3	0.035	Case–control/cross-sectional	3	1.78 (1.13–2.79)	83.0	0.003
Cohort	2	0.81 (0.61–1.07)	0.0	0.677	Cohort	2	1.16 (0.80–1.68)	0.0	0.648
Study area					Study area				
Western countries	3	0.88 (0.68–1.13)	14.8	0.309	Western countries	3	1.42 (0.93–2.17)	42.3	0.177
Other countries	2	0.59 (0.42–0.82)	26.3	0.244	Other countries	2	1.65 (0.94–2.91)	90.8	0.001
Sample size					Sample size				
≥5,000	3	0.88 (0.68–1.13)	14.8	0.309	>5,000	3	1.42 (0.93–2.17)	42.3	0.177
<5,000	2	0.59 (0.42–0.82)	26.3	0.244	<5,000	2	1.65 (0.94–2.91)	90.8	0.001
Dietary assessment method					Dietary assessment method				
FFQ	3	0.73 (0.57–0.94)	41.6	0.180	FFQ	3	1.52 (0.92–2.51)	75.7	0.016
Other	2	0.81 (0.58–1.12)	72.9	0.055	Other	2	1.55 (0.88–2.73)	67.8	0.078

RR: relative risk; CI: confidence interval; FFQ: Food frequency questionnaire.

#### Western dietary pattern and Parkinson’s disease

**Figure 5:**
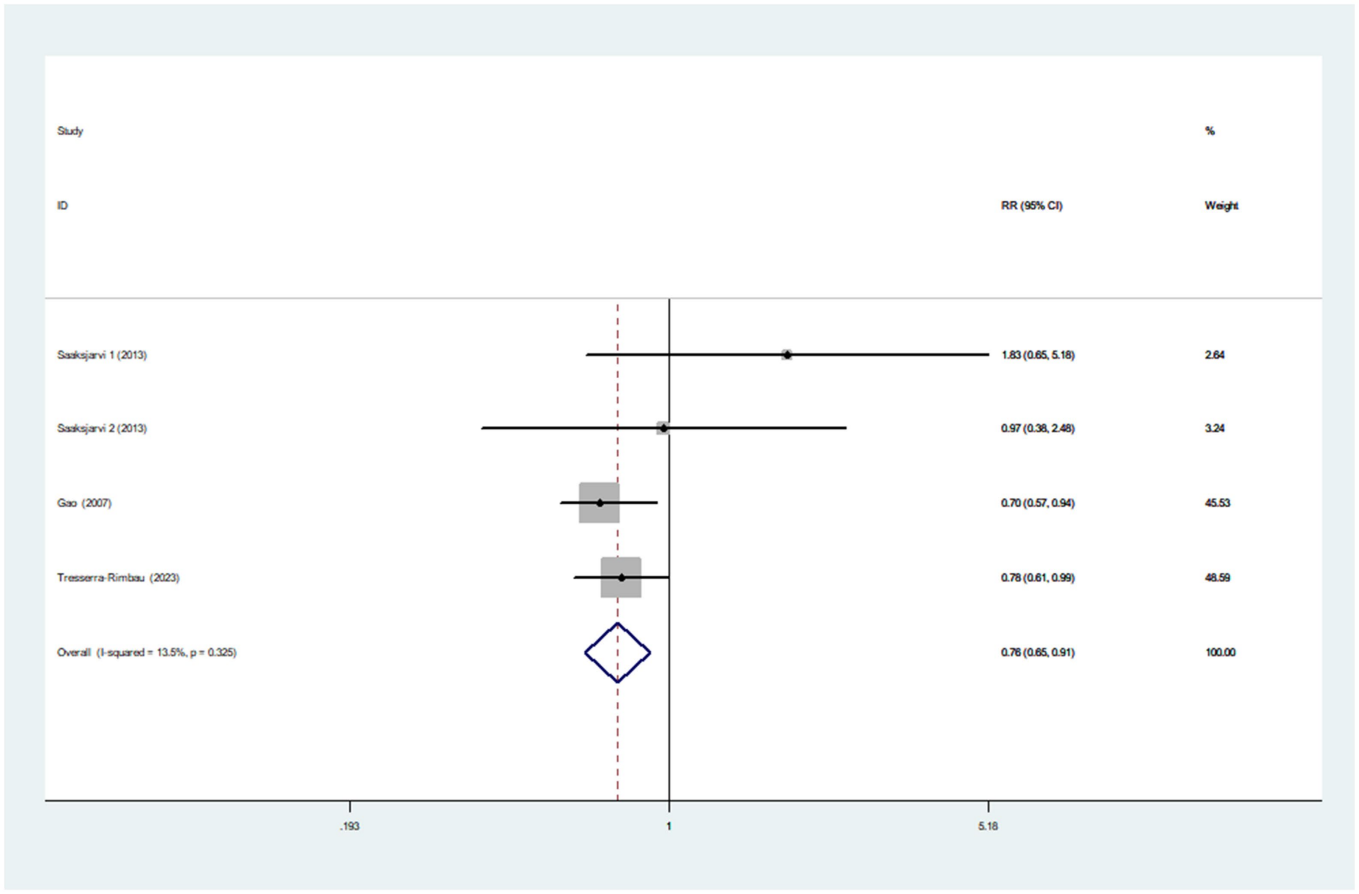
Forest plot of the association between Western dietary pattern and risk of Parkinson’s disease.

Pooled results from five articles identified the Western dietary pattern. Figure 5 showed the obvious evidence of an increased risk of Parkinson’s disease in the highest compared with lowest categories of Western dietary pattern (RR = 1.54; 95%CI: 1.10–2.15; *p =* 0.011). There was significant heterogeneity in the included studies (*p* = 0.004; *I*^2^ = 73.5%), and thus a random-effects model was used to calculate the combined RR. In our analyses, subgroup analyses were carried out based on study design, study area, sample size and dietary assessment method. The results showed that study design might explain, to some extent, the significant heterogeneity across studies (Table 4).

### Quality assessment

The quality of included studies using NOS criteria is shown in Table 5. Based on NOS tool, eight article receiving a score of seven or higher, were considered to be of high quality (14–20, 22, 24). Additionally, the remaining two articles were identified as medium-quality (21, 23).

**Table 5:** Priori and posteriori dietary patterns and risk of Parkinson’s disease: Assessment of Study Quality.

Studies	Selection				Comparability		Outcome			Score
	1	2	3	4	5A	5B	6	7	8	
Cohort										
Gao et al. (2007) (14)	*	*	*	*	*	*	*	*	*	9
Yin et al. (2021) (17)	*	*	*	*	*	*	*	*	*	9
Tresserra-Rimbau et al. (2023) (19)	*	*	*	*	*	*	*	*	*	9
Strikwerda et al. (2021) (20)	*	*	*	*	*	*	*	*	*	9
Sääksjärvi et al. (2013) (24)	*	*	*	*	*		*	*	*	8
Case–control										
Alcalay et al. (2012) (15)	*	*	*		*	*	*	*	*	8
Okubo et al. (2012) (16)	*	*	*		*	*	*	*		7
Shokri-Mashhadi et al. (2024) (22)	*	*	*		*	*	*	*		7
Cross-sectional										
Xu et al. (2023) (18)	*	*	*	*	*		*	*		7
Keramati et al. (2023) (21)	*	*	*		*		*	*		6
Agarwal et al. (2018) (23)	*	*	*		*		*	*		6

*For case–control studies, 1 indicates cases independently validated; 2, cases are representative of population; 3, community controls; 4, controls have no history of Parkinson’s disease; 5A, study controls for the most important factor; 5B, study controls for additional factors, e.g., cigarette smoking body mass index, total energy intake; 6, ascertainment of exposure by secure record or blinded interview or record; 7, same method of ascertainment used for cases and controls; and 8, the same for cases and controls. For cohort studies, 1 indicates exposed cohort truly representative; 2, non exposed cohort drawn from the same community; 3, ascertainment of exposure by secure record (e.g., surgical records) or structured interview; 4, outcome of interest was not present at start of study; 5A, study controls for the most important factor; 5B, study controls for additional factor (s); 6, assessment of outcome is based on independent blind assessment or record linkage; 7, follow-up long enough(≥5 years) for outcomes to occur; and 8, adequacy of follow up of cohorts (all participants complete follow up or >90% participants complete follow up).

### Publication bias and sensitivity analyses

Visual inspection of the funnel plots indicated no evidence of asymmetry (Supplementary Figures 1–4). Similarly, Egger’s and Begg’s tests for publication bias were not statistically significant (highest compared with lowest category of Mediterranean diet: Begg’s test, *p* = 0.764; Egger’s test, *p* = 0.043; Healthy dietary index: Begg’s test, *p* = 0.308; Egger’s test, *p* = 0.161; Healthy dietary pattern: Begg’s test, *p* = 1.000; Egger’s test, *p* = 0.943; Western pattern: Begg’s test, *p* = 0.462; Egger’ s test, *p* = 0.860). Based on the results of sensitivity analyses, no significant changes in pooled RRs were found after eliminating any single or a few studies of both *a priori* and a posteriori dietary patterns in respect to risk of Parkinson’s disease (Supplementary Figures 5–10).

## Discussion

The existing literature regarding the associations between dietary patterns and risk of Parkinson’s disease are limited and controversial. As far as we are aware, this is the latest and most comprehensive systematic review and meta-analysis evaluating the associations between a priori and a posteriori dietary patterns and Parkinson’ s disease. This meta-analysis demonstrated that adherence to the Mediterranean diet, healthy dietary index and healthy dietary pattern were associated with a reduced risk of Parkinson’s disease, whereas the Western dietary pattern was linked to an increased risk of Parkinson’s disease. Given the significant heterogeneity observed in this meta-analysis, these results should be interpreted with caution. Collectively, our findings provide substantive evidence for the significant associations between dietary patterns and Parkinson’s disease risk, and support the adoption of whole dietary patterns for the prevention of Parkinson’s disease.

### Comparison with epidemiological studies

Over the past few decades, Parkinson’s disease has become the second most common neurodegenerative disease worldwide, and its incidence has steadily increased (6, 33). Additionally, studies have shown that the economic burden of Parkinson’s disease in the United States is expected to increase from $52 billion in 2017 to $79 billion in 2037 (4). Considering the tremendous cost on public health, it is essential to explore the modifiable dietary factors for the prevention of Parkinson’s disease. Previous studies have shown the associations between whole dietary patterns (e.g., Mediterranean diet, the Alternative healthy eating index, and prudent pattern) and Parkinson’s disease risk (14–24). Nevertheless, it was noteworthy that these studies yielded inconsistent results. In the Health Professionals Follow-Up Study and the Nurses’ Health Study, Gao et al. found that higher adherence to the alternate Mediterranean diet score was inversely associated with Parkinson’s disease risk (HR = 0.75; 95%CI: 0.57–1.00) (14). However, at variance with the aforementioned study, Strikwerda and colleagues observed no significant association between adherence to the Mediterranean diet and Parkinson’s disease risk in the prospective population-based cohort study (HR = 0.80; 95%CI: 0.50–1.29) (20). The reasons for the inconsistent results may be attributed to differences in study design, sample size, study population, and dietary assessment method. In this study, we found that higher adherence to the Mediterranean diet, healthy dietary index and healthy dietary pattern were associated with a lower risk of Parkinson’s disease. Similar to our meta-analysis, a recent systematic review and meta-analysis conducted in 2024 demonstrated a significant negative correlation between adherence to Mediterranean diet and Parkinson’s disease (25). Notably, in Zhao’s meta-analysis, the outcome of interests included Parkinson’s disease, prodromal Parkinson’s disease and prodromal Parkinson’s disease features. Moreover, a hospital-based case–control study in Japan that reported an association between healthy pattern and Parkinson’s disease (16) has also been included in their analyses. Furthermore, the aforementioned meta-analysis only reported an association between Mediterranean diet adherence and Parkinson’s disease, and did not report the associations between a posteriori dietary patterns and risk of Parkinson’s disease. In this context, we performed this systematic review and meta-analysis of observational studies to determine the role of *a priori* and a posteriori dietary patterns in Parkinson’s disease.

While epidemiological evidence regarding the links between the Mediterranean diet, healthy dietary index, and healthy dietary pattern and Parkinson’s disease risk remains inconclusive, a number of possible mechanisms have been proposed to explain the observed associations. First, it is well-known that the Mediterranean diet, healthy dietary index and healthy dietary pattern emphasize a high consumption of vegetables, fruits, nuts, legumes and whole grains. The protective effect of vegetables and fruits on Parkinson’s disease may be related to high concentration of antioxidants. Earlier studies have shown an inverse association between Parkinson’s disease risk and intake of antioxidants, such as vitamin C, vitamin E and carotenoids (34). In parallel, a recent systematic review and dose–response meta-analysis of observational studies also showed that higher intake of antioxidant-rich foods was associated with a lower risk of Parkinson’s disease (35). Second, all three dietary patterns mentioned above are considered as plant-based diets. Previous studies have shown that plant-based diets can reduce inflammation (22), which is an important mechanistic link in neurodegenerative diseases, including Parkinson’s disease (36). Third, legumes that contain procyanidin (an important food component in a healthy diet), are high in magnesium and have a low glycemic index, which is linked to the reduced oxidative stress (37). Indeed, neuroinflammation caused by oxidative stress is known to be implicated in the pathogenesis of Parkinson’s disease (38). Fourth, vegetables, fruits and whole grains are rich in dietary fiber, which may mitigate gut dysbiosis by favoring beneficial gut bacteria (39). Studies have shown that gut dysbiosis, an alteration of the gut microbiome, has been suggested as a mechanism by which neuroinflammation leads to Parkinson’s disease (40). Also, dietary fiber produces short-chain fatty acids, which play an anti-inflammatory role and delay the progression of Parkinson’s disease (41). As already discussed above, these mechanisms could together explain the favorable associations observed between adherence to the Mediterranean diet, healthy dietary index, and healthy dietary pattern and risk of Parkinson’s disease.

As reported in Figure 5, Western dietary pattern was associated with an increased risk of Parkinson’s disease. Our results were in agreement with previous studies (22), which suggested that adherence to western dietary pattern could increase the risk of Parkinson’s disease. To our knowledge, there are several plausible explanations associating Western dietary pattern with increased risk of Parkinson’s disease. First, previous research has suggested that consumption of sweeteners and processed foods may lead to changes in the composition and function of the gut microbiota, which may contribute to the development of inflammation (42). As mentioned previously, inflammation is an important mechanistic link in the development of Parkinson’s disease (36). Moreover, high intake of sugar has been shown to increase insulin resistance, a potential risk factor for Parkinson’s disease (43). Second, Western dietary pattern was characterized by low intake of dietary fiber. Evidence suggests that low dietary fiber intake favors the growth of Gram-negative bacterial, which can lead to neurodegenerative diseases (44). Third, red meat often contains high content of iron. A previous meta-analysis showed that higher intake of iron was associated with an increased risk of Parkinson’s disease in Western population (45). Additionally, Jiang and colleagues found that dietary iron intake was positively associated with insulin resistance (46), an important risk factor for Parkinson’s disease (43). Fourth, high-fat dairy products, French fries, desserts and red meat are the main food components of Western dietary pattern, containing high amounts of saturated fat. Epidemiological studies have shown that high intake of saturated fatty acids was associated with increased risk of Parkinson’s disease (47).

In our analyses, the results showed substantial heterogeneity in the associations between adherence to the Mediterranean diet (I^2^ = 64.3%, *p* = 0.010) and Western dietary pattern (*p* = 0.004; *I*^2^ = 73.5%) and risk of Parkinson’s disease. Heterogeneity between studies has been common in previous meta-analyses (11), but there is a need to explore potential sources of substantial heterogeneity. In this study, we carried out subgroup analyses by study design, study area, mean age, sample size, study quality and dietary assessment method. For the Mediterranean diet, the results showed that differences in study design, sample size and study quality might contribute to substantial heterogeneity. When subgroup analyses were performed for study design, sample size and study quality separately, heterogeneity decreased from 64.3 to 0.0%. For Western dietary pattern, the results showed that difference in study design might partly explain the significant heterogeneity. Similarly, when subgroup analysis was performed for study design, heterogeneity decreased from 73.5 to 0.0%. Although significant heterogeneity cannot be fully explained by differences in the above variables, there are several possible explanations for significant heterogeneity. First, recall bias resulting from dietary assessment methods in the eligible studies, including FFQs and dietary questionnaire, should be noted. Second, because all included studies were observational, the results may have been affected by residual or unmeasured factors. Third, the included studies used inconsistent adjusted variables, which could explain the significant heterogeneity. Fourth, given the differences in the definition of Mediterranean diet in different countries, dietary habits may lead to significant heterogeneity. Fifth, our analyses did not use the pre-registering search methods, which may have led to bias. Finally, significant heterogeneity still persisted in the subgroup analyses, suggesting the presence of other unmeasured confounding factors.

### Strengths and limitations

There are numerous strengths in this study. First, this is the latest and most comprehensive systematic review and meta-analysis evaluating the associations between *a priori* and a posteriori dietary patterns and Parkinson’ s disease. Compared with previous meta-analyses, we performed a rigorous article screening and excluded several articles on dietary patterns and prodromal Parkinson’s disease. Additionally, our findings provide some evidence for the associations between whole dietary patterns and Parkinson’s disease risk, and emphasize the importance of adherence to the healthy dietary patterns for the prevention of Parkinson’ s disease. Second, Parkinson’ s disease cases have been identified by hospital records or a neurologist, avoiding misdiagnosis bias. Third, subgroup and sensitivity analyses were carried out to explore the possible sources of heterogeneity between studies, improving the accuracy of the results. Fourth, the funnel plots and Begg’s and Egger’s tests did not show any significant asymmetry, suggesting a low risk of publication bias. Despite these strengths, some limitations should be considered when interpreting the results of our meta-analysis. First of all, the observational design of all eligible studies precludes us from establishing a causal association between *a priori* and a posteriori dietary patterns and risk of Parkinson’ s disease. Second, the large majority of included studies used FFQs to collect dietary data, which may lead to the under- or over- estimation of healthy foods. Third, although all included studies attempted to control or adjust for various potentially confounding variables, the possibility of residual confounding cannot be excluded. Fourth, we could not perform a dose–response analysis, due to insufficient data reported in the included studies. Fifth, the protocol of this study was not registered online in advance with PROSPERO database, but we strictly followed the PRISMA guidelines to reduce selection bias. Additionally, since we did not search for grey literature or unpublished studies, selective reporting bias could not be ruled out in this study. Sixth, only three studies reported the association between healthy dietary index and Parkinson’s disease, preventing us from performing a subgroup analysis. Finally, significant heterogeneity was observed in our analyses. Even though we performed subgroup and sensitivity analyses to explore the potential sources of heterogeneity, we could not ascertain and explain the sources of inter-study heterogeneity sufficiently.

## Conclusion

In conclusion, the results of this study showed that adherence to the Mediterranean diet, a healthy dietary index and a healthy dietary pattern were associated with a reduced risk of Parkinson’s disease, whereas the Western dietary pattern was linked to an increased risk of Parkinson’s disease. Our findings add the current evidence and emphasize the importance of adopting healthy patterns, such as prudent pattern and Mediterranean diet for the prevention of Parkinson’s disease. As epidemiological evidence on this topic is limited, additional prospective studies and randomized controlled trials are urgently required to corroborate these findings.

## Data Availability

The datasets presented in this study can be found in online repositories. The names of the repository/repositories and accession number(s) can be found in the article/Supplementary material.
