# Supplementary figures and images for: Adherence to a priori and a posteriori dietary patterns and risk of Parkinson’s disease: a systematic review and meta-analysis of observational studies
Source: Front Nutr. 2025 May 12;12:1600955. doi: 10.3389/fnut.2025.1600955 (PMC12105580; doi:10.3389/fnut.2025.1600955)

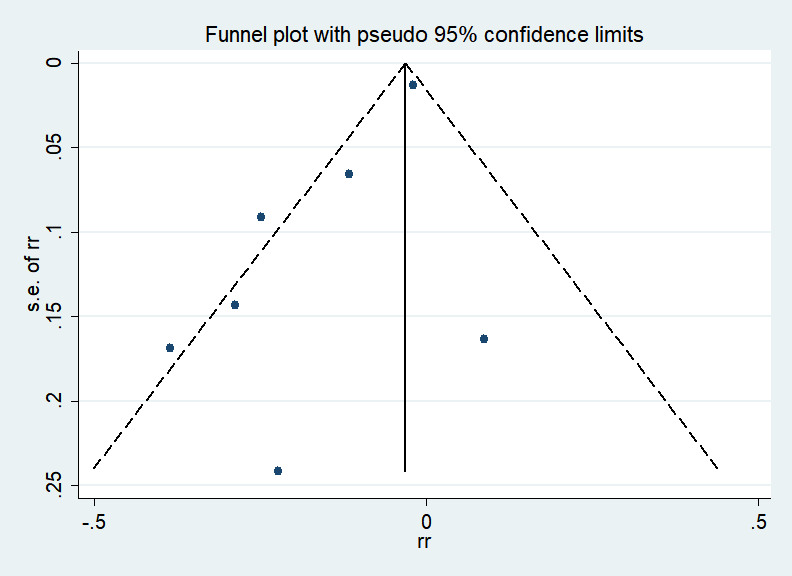

Supplement: Supplementary file 1 [file Image_1.TIF]

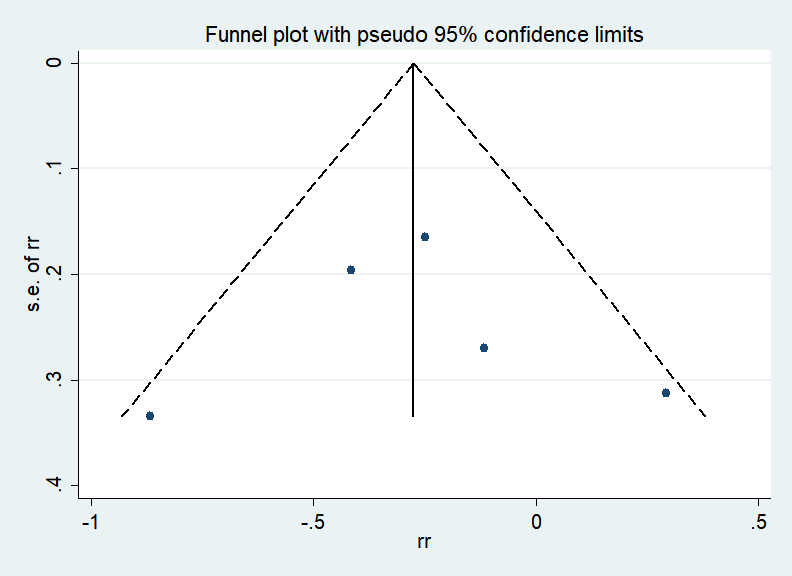

Supplement: Supplementary file 2 [file Image_2.TIF]

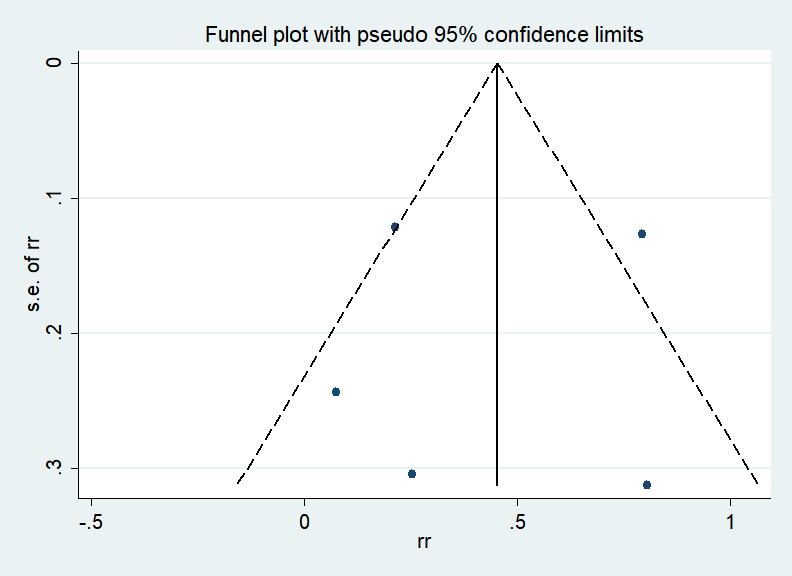

Supplement: Supplementary file 3 [file Image_3.TIF]

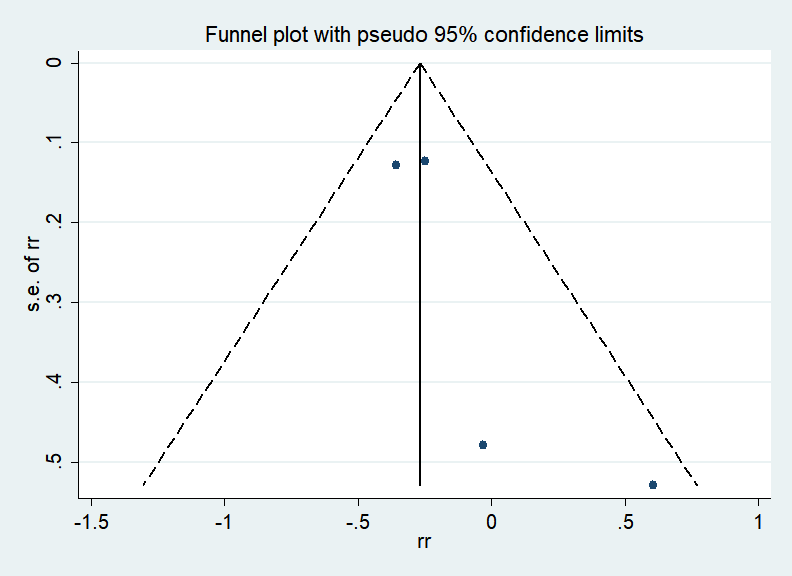

Supplement: Supplementary file 4 [file Image_4.TIF]

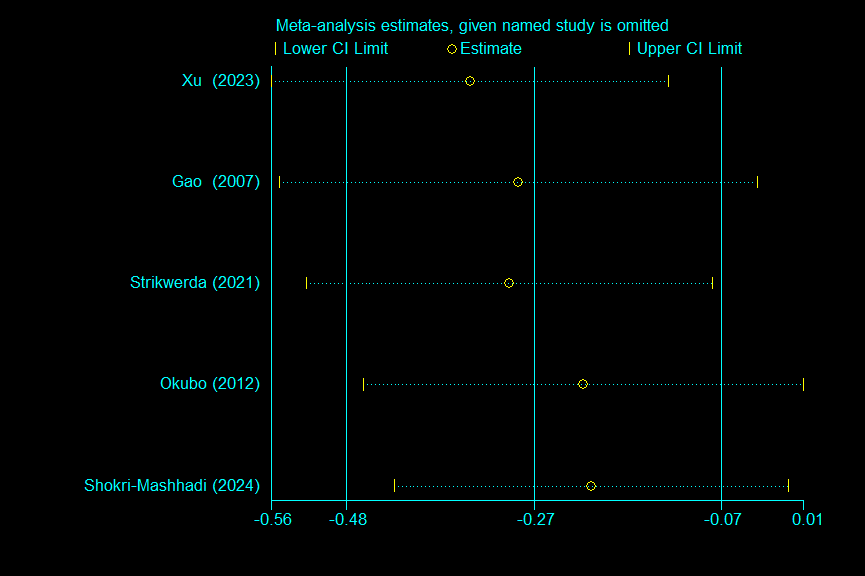

Supplement: Supplementary file 5 [file Image_5.TIF]

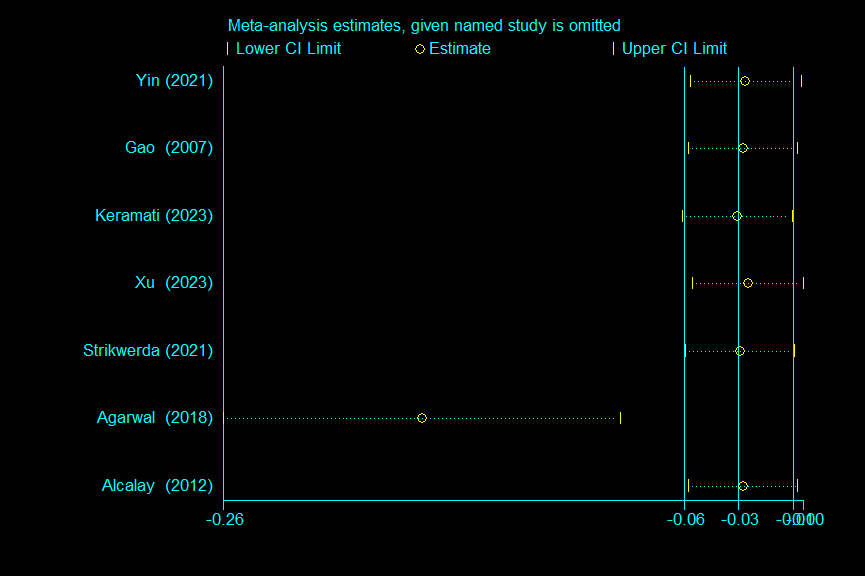

Supplement: Supplementary file 6 [file Image_6.TIF]

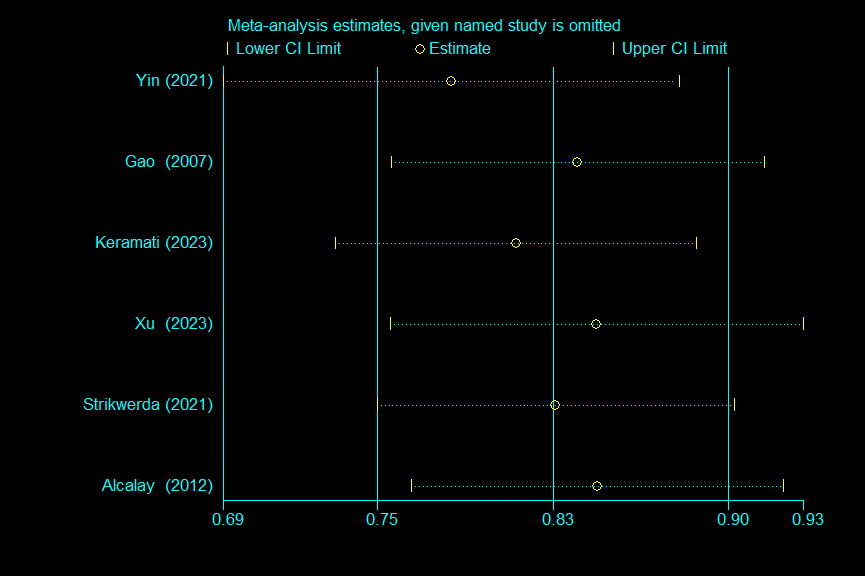

Supplement: Supplementary file 7 [file Image_7.TIF]

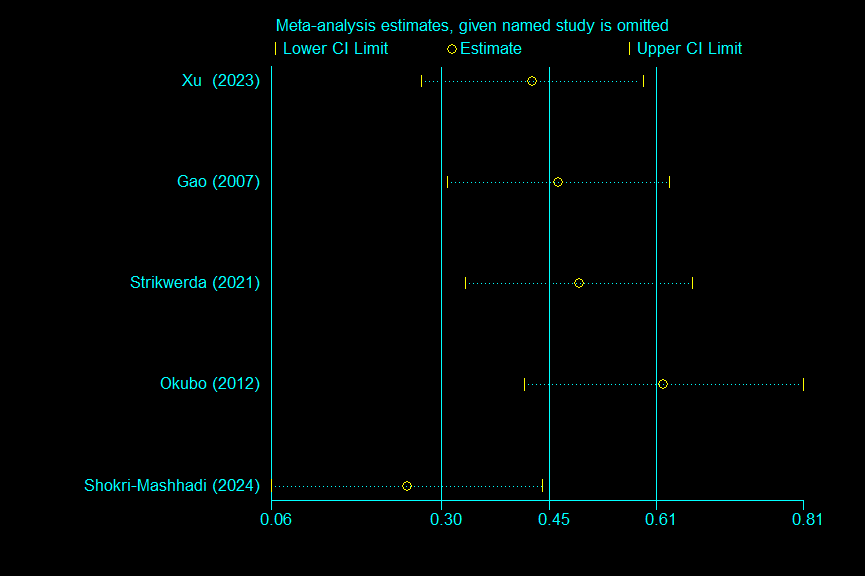

Supplement: Supplementary file 8 [file Image_8.TIF]

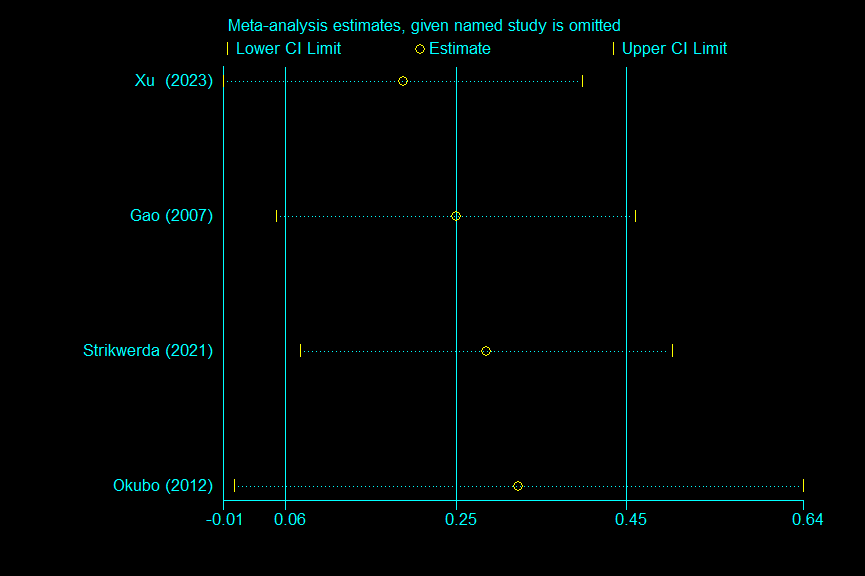

Supplement: Supplementary file 9 [file Image_9.TIF]

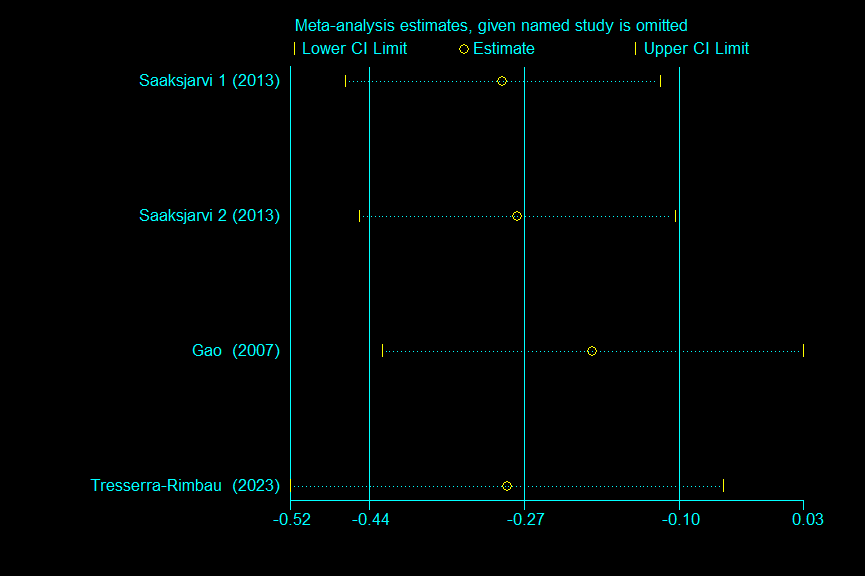

Supplement: Supplementary file 10 [file Image_10.TIF]
